# High mRNA Expression of *CENPL* and Its Significance in Prognosis of Hepatocellular Carcinoma Patients

**DOI:** 10.1155/2021/9971799

**Published:** 2021-08-18

**Authors:** Zhongyuan Cui, Lijia Xiao, Fengsui Chen, Jielong Wang, Haiyan Lin, Dongliang Li, Zhixian Wu

**Affiliations:** ^1^Department of Hepatobiliary Disease, 900th Hospital of the Joint Logistics Support Force, Dongfang Hospital, Xiamen University, Fuzhou, Fujian 350025, China; ^2^Department of Disease Prevention and Control, General Hospital of Western Theater Command, Chengdu, Sichuan 610000, China; ^3^Department of Hepatobiliary Disease, 900th Hospital of the Joint Logistics Support Force, Fujian Medical University, Fuzhou, Fujian 350025, China

## Abstract

Centromere proteins (CENPs) are the main constituent proteins of kinetochore, which are essential for cell division. In recent years, several studies have revealed that several CENPs were aberrantly expressed in hepatocellular carcinoma (HCC). However, numerous centromere proteins have not been studied in HCC. In this study, we used databases of Oncomine, Gene Expression Profiling Interactive Analysis (GEPIA), the Kaplan-Meier Plotter, cBioPortal, the Human Protein Atlas (HPA), and TIMER (Tumor Immune Estimation Resource) and immunohistochemical staining of clinical specimens to investigate the expression of 15 major centromere proteins in HCC to evaluate their potential prognostic value. We found that the mRNA levels of 4 out of 15 centromere proteins (CENPL, CENPQ, CENPR, and CENPU) were significantly higher in HCC than in normal tissues, and their mRNA levels were associated with the tumor stages (*p* values < 0.01). Patients with higher mRNA levels of *CENPL* had poorer overall survival, progression-free survival, relapse-free survival, and disease-specific survival (*p* values < 0.05). Furthermore, the higher levels of *CENPL* mRNA were associated with worse overall survival in males without hepatitis virus infection (*p* values < 0.05). The protein expression level of CENPL in human HCC tissue was higher than that in normal liver tissue. In addition, the expression of *CENPL* was positively correlated with the levels of the tumor-infiltrating lymphocytes. The results suggest that the high mRNA expression of *CENPL* may be a potential predictor of prognosis in HCC patients.

## 1. Introduction

Hepatocellular carcinoma (HCC) is the fourth major cause of cancer-related death globally [1]. Patients with early-stage HCC are eligible to local ablation, surgical resection, or liver transplantation. Unfortunately, due to the lack of satisfactory biomarkers in diagnosis, treatment, and prognosis, morbidity and mortality continue to increase [2]. Therefore, it is necessary to explore novel diagnostic, therapeutic, and prognostic biomarkers for HCC.

The centromere proteins (CENPs) are assembled in repetitive noncoding DNA regions (centromere DNA). They are named in alphabetical order, according to the date of identification [3]. To date, more than 100 centromere proteins have been identified. These proteins are attached to chromosomes and spindle microtubules and are involved in precise regulation and guidance of chromosome separation during mitosis or meiosis [3, 4] A growing number of studies have revealed that several CENPs were abnormally expressed in lung, breast, ovarian, bladder, esophageal, and colorectal cancers and were correlated with the prognosis of patients [5–14].

Several members of CENPs have been reported to be involved in HCC. These studies showed that *CENPA*, *CENPE*, *CENPF*, *CENPH*, *CENPK*, and *CENPW* were all abnormally expressed in HCC and were associated with patient prognosis [15–29]. Current studies have suggested that these CENPs might play an important role in HCC, and many others remained unknown. With the advancement in bioinformatics, a variety of high-quality databases are available for discovering novel neoplastic factors [30–32]. Based on the public databases, 15 major CENPs, including *CENPB*, *CENPC*, *CENPI*, *CENPJ*, *CENPL*, *CENPN*, *CENPO*, *CENPP*, *CENPQ*, *CENPR*, *CENPS*, *CENPT*, *CENPU*, *CENPV*, and *CENPX* were analyzed by using bioinformatics tools and immunohistochemical staining to further investigate the gene expression patterns and prognostic value in HCC.

## 2. Methods

### 2.1. Oncomine Database Analysis

We used Oncomine (http://www.oncomine.org), an online cancer microarray database [33], to analyze the transcription levels of 15 CENPs in tumors and normal tissues from HCC and other cancers. Datasets of clinical tumor and normal control specimens were compared using Student's *t-*test. For all cancers and genes, the threshold was set as the *p* value of 0.0001 and the fold change of 2.

### 2.2. GEPIA Dataset Analysis

Gene Expression Profiling Interactive Analysis (GEPIA) (http://gepia.cancer-pku.cn) is a newly developed interactive web server for analyzing the RNA sequencing expression data from the TCGA and the GTEx through a variety of customizable functions [34]. It was used to analyze mRNA differential expression in HCC and liver tissues, as well as the correlation between expression levels and pathological stages. Differential threshold of ∣log_2_FC∣ was 1 and the *p* value cutoff of 0.01.

### 2.3. The Kaplan-Meier Plotter

The Kaplan-Meier Plotter (http://www.kmplot.com) database was used to explore the relationship between gene transcription and prognosis. The database contains the levels of CENP mRNAs and survival information including OS, progression-free survival (PFS), RFS, and disease-specific survival (DSS) under various clinical parameters of HCC patients (https://kmplot.com/analysis/index.php?P=service&cancer=liver_rnaseq) [35]. The patients were divided into two groups based on the median mRNA expression of CENPs. The *p* value of <0.05 is considered significantly different.

### 2.4. cBioPortal Database

cBioPortal (https://www.cbioportal.org/) is a comprehensive gene database, including different datasets such as DNA mutation, gene amplification, and methylation. This database was used to analyze and visualize possible gene mutation and copy number alterations of CENPs.

### 2.5. TIMER

TIMER (Tumor Immune Estimation Resource) is a web server for comprehensive analysis of tumor-infiltrating immune cells (https://cistrome.shinyapps.io/timer/). The relationships between the expression levels of potential CENPs and 6 immune infiltrates (B cells, CD4+ T cells, CD8+ T cells, neutrophils, macrophages, and dendritic cells) were estimated by the TIMER algorithm in HCC. Moreover, the correlation of tumor purity and CENP gene expression was also analyzed. *p* < 0.05 is considered as significant.

### 2.6. The Human Protein Atlas

The Human Protein Atlas (HPA) is a Swedish-based program initiated in 2003, which is aimed at mapping all the human proteins in cells, tissues, and organs using an integration of various omics technologies, including antibody-based imaging, mass spectrometry-based proteomics, transcriptomics, and systems biology (https://www.proteinatlas.org/). The Human Pathology Atlas is based on a systems-based analysis of the transcriptome of 17 main cancer types using data from 8000 patients [36]. In this database, we verified the protein expression of CENPs with potential prognostic value in normal liver and HCC tissues.

### 2.7. Immunohistochemical Staining

Five *μ*m sections were obtained from paraffin-embedded tumor and nontumor specimens from 28 patients with clinically diagnosed HCC. All these sections were dewaxed in xylene and rehydrated in alcohol followed by wet autoclave pretreatment in citrate buffer for antigen retrieval (10 minutes at 120°C, pH = 6.0). Then, the sections were rinsed with phosphate buffer saline. Immunohistochemical staining for antibody to CENPL (rabbit: bs13836R, Beijing Biosynthesis Biotechnology, China) was performed using the avidin-biotin-peroxidase complex method. The primary antibody was applied to the sections and allowed to react for 1 hour at room temperature. The sections were then incubated with biotinylated anti-mouse/rabbit antibody (1 : 200 dilution for CENPL) for 30 min and avidin-biotin-peroxidase reagent for 25 min. After color development with diaminobenzidine, the sections were counterstained with hematoxylin.

## 3. Results

### 3.1. The Transcription Levels of 15 CENPs in Hepatocellular Carcinoma

**Figure 1:**
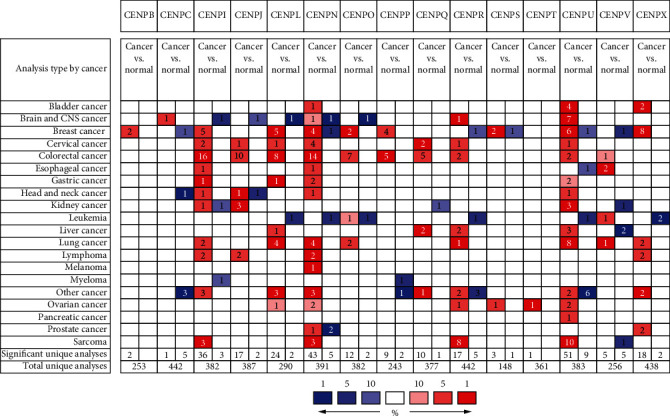
The transcription levels of CENP in different types of cancers. The mRNA levels of *CENPL*, *CENPQ*, *CENPR*, and *CENPU* were significantly higher in HCC than in normal tissues (*p* < 0.01) (Oncomine).

**Figure 2:**
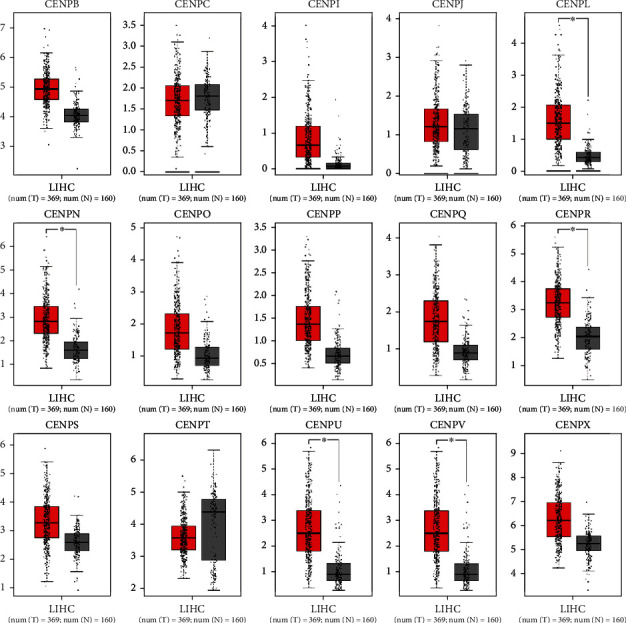
The expression of CENP mRNA levels in HCC. The transcript levels of *CENPB*, *CENPI*, *CENPL*, *CENPN*, *CENPO*, *CENPP*, *CENPQ*, *CENPR*, *CENPS*, *CENPU*, *CENPV*, and *CENPX* were significantly higher in the tumor than in the normal tissue (*p* < 0.01) (GEPIA, box plot).

**Figure 3:**
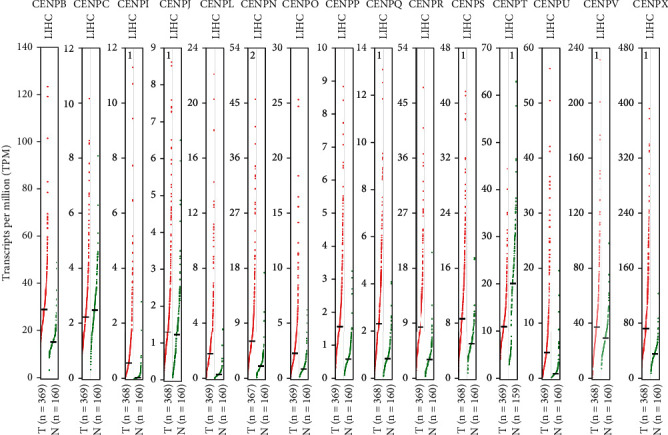
The expression of CENP mRNA levels in HCC. The transcript levels of *CENPB*, *CENPI*, *CENPL*, *CENPN*, *CENPO*, *CENPP*, *CENPQ*, *CENPR*, *CENPS*, *CENPU*, *CENPV*, and *CENPX* were significantly higher in the tumor than in the normal tissue (*p* < 0.01) (GEPIA, scatter diagram).

We investigated the mRNA levels of 15 CENPs in the tumor and normal tissues of HCC and other major cancers by using Oncomine database. We found that 4 out of 15 CENPs (*CENPL*, *CENPQ*, *CENPR*, and *CENPU*) were significantly overexpressed in HCC (Figure 1). Fold change ranged from 2.035 to 4.861 (Table 1). *CENPV* was underexpressed in two dysplasia datasets with fold change of -2.075 and -2.392 (Table 1). However, no significant change in 10 CENPs (*CENPB*, *CENPC*, *CENPI*, *CENPJ*, *CENPN*, *CENPO*, *CENPP*, *CENPS*, *CENPT*, and *CENPX*) were observed (Figure 1). These datasets were reported by Wurmbach et al. [37], Roessler et al. [38], and Chen et al. [39], respectively. The results of GEPIA showed that the mRNA levels of 12 CENPs (*CENPB*, *CENPI*, *CENPL*, *CENPN*, *CENPO*, *CENPP*, *CENPQ*, *CENPR*, *CENPS*, *CENPU*, *CENPV*, and *CENPX*) were significantly higher in the tumor than in the normal tissue (Figures 2 and 3).

### 3.2. Relationship between the mRNA Levels of CENPs and Pathological Stages of HCC

**Figure 4:**
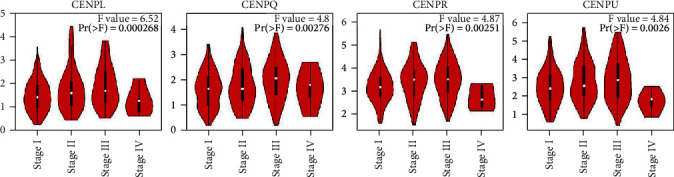
Correlation between CENP mRNA levels and tumor stages in HCC patients. The mRNA levels of *CENPL*, *CENPQ*, *CENPR*, and *CENPU* were significantly correlated with tumor stages (*p* < 0.01). (GEPIA).

Then, we selected 4 CENPs (*CENPL*, *CENPQ*, *CENPR*, and *CENPU*) with high expression in HCC as shown in both the Oncomine and GEPIA databases to further analyze the relationship between mRNA levels and tumor stages. The analysis showed that high mRNA levels of *CENPL*, *CENPQ*, *CENPR*, and *CENPU* were significantly correlated with tumor stages (*p* < 0.01) (Figure 4).

### 3.3. Correlation between the Increased mRNA Levels and the Prognosis of CENPs in Patients with HCC

**Figure 5:**
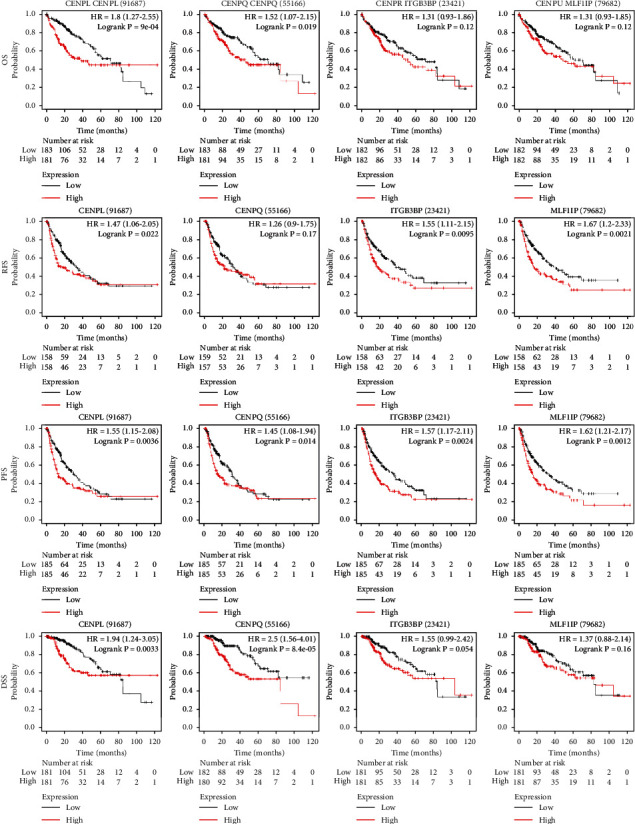
Correlation between increased CENP mRNA level (*CENPL*, *CENPQ*, *CENPR*, and *CENPU*) and prognosis in patients with HCC. Patients with higher mRNA levels of *CENPL* had significantly poorer OS, PFS, RFS, and DSS (*p* < 0.05) (the Kaplan-Meier Plotter).

To evaluate the prognostic value of 4 CENPs (*CENPL*, *CENPQ*, *CENPR*, and *CENPU*) with high mRNA expression in HCC patients, we used the Kaplan-Meier Plotter to analyze their overall survival (OS), progression-free survival (PFS), relapse-free survival (RFS), and disease-specific survival (DSS). It was found that patients with higher mRNA levels of *CENPL* were associated with significantly poor OS, PFS, RFS, and DSS (*p* < 0.05) (Figure 5, Table 2). Patients with higher *CENPQ* mRNA expression had poorer OS, PFS, and DSS (*p* < 0.05), and patients with higher *CENPR* mRNA levels had poorer PFS and RFS (*p* < 0.05). Patients with increased *CENPU* mRNA levels had lower PFS and RFS (*p* < 0.05) (Figure 5, Table 2).

**Figure 6:**
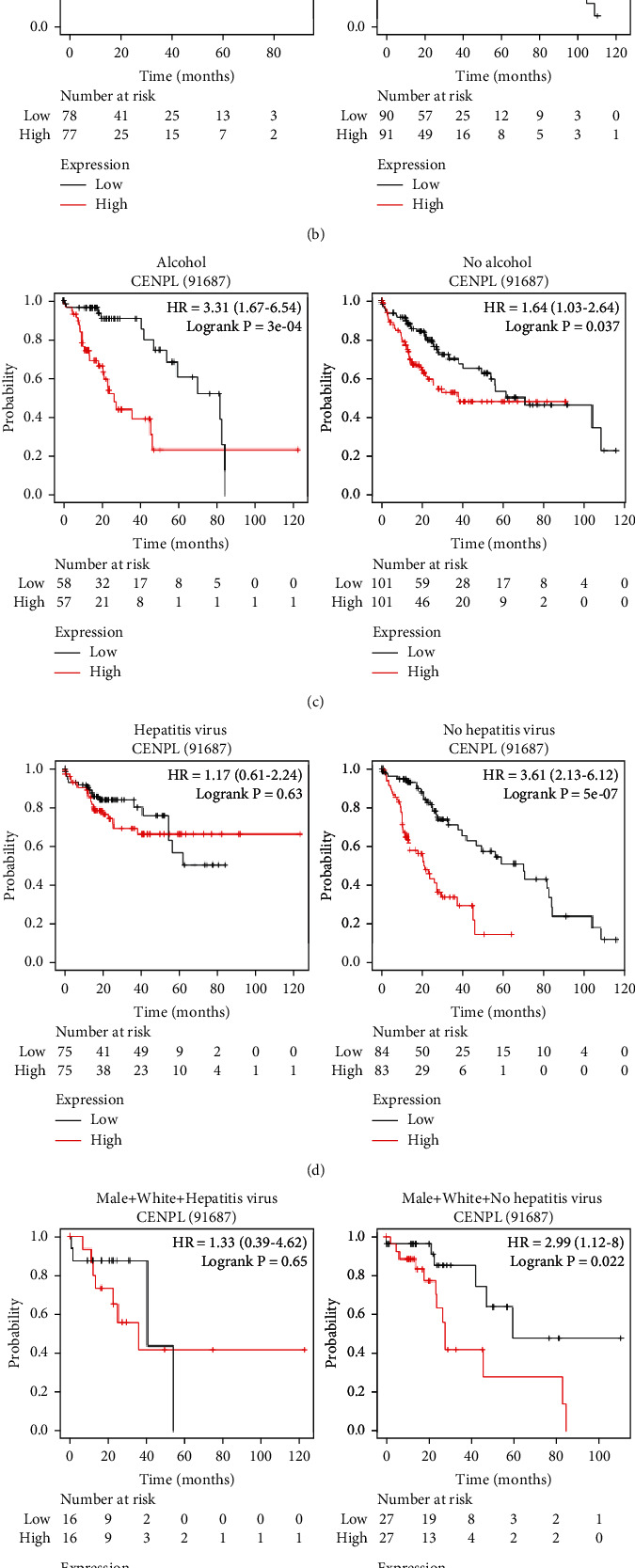
Correlationship between increased CENPL mRNA levels and patients' OS with various clinical parameters. Males with higher mRNA level of CENPL had significantly poorer OS (a). Asians with higher mRNA levels of CENPL had worse OS (b). Patients with higher *CENPL* mRNA level had poorer OS regardless of alcohol consumption (c). Patients without hepatitis virus infection had poorer OS with high expression of *CENPL* (d). Among male patients without hepatitis virus infection, white and Asian patients with higher *CENPL* mRNA level had poorer OS (e, f) (the Kaplan-Meier Plotter).

The level of *CENPL* mRNA showed a potential value in predicting the prognosis of patients with HCC. Therefore, we further explored the relationship between the mRNA expression of CENPL and the OS of patients in subgroups as divided by parameters including gender, race, alcohol consumption, and hepatitis virus infection. The results showed that males with high expression of CENPL mRNA had poorer OS (Figure 6(a), Table 3). Asians with higher mRNA levels of CENPL had worse OS (Figure 6(b), Table 3). Patients with higher CENPL expression had worse OS regardless of alcohol consumption (Figure 6(c), Table 3). Among patients without hepatitis virus infection, patients with higher expression of *CENPL* had poorer OS (Figure 6(d), Table 3). Furthermore, we found that higher levels of *CENPL* mRNA were associated with worse OS in white and Asian male patients without hepatitis virus infection (Figures 6(e) and 6(f), Table 3).

### 3.4. Mutations and Copy Number Alterations of CENPL in HCC

**Figure 7:**
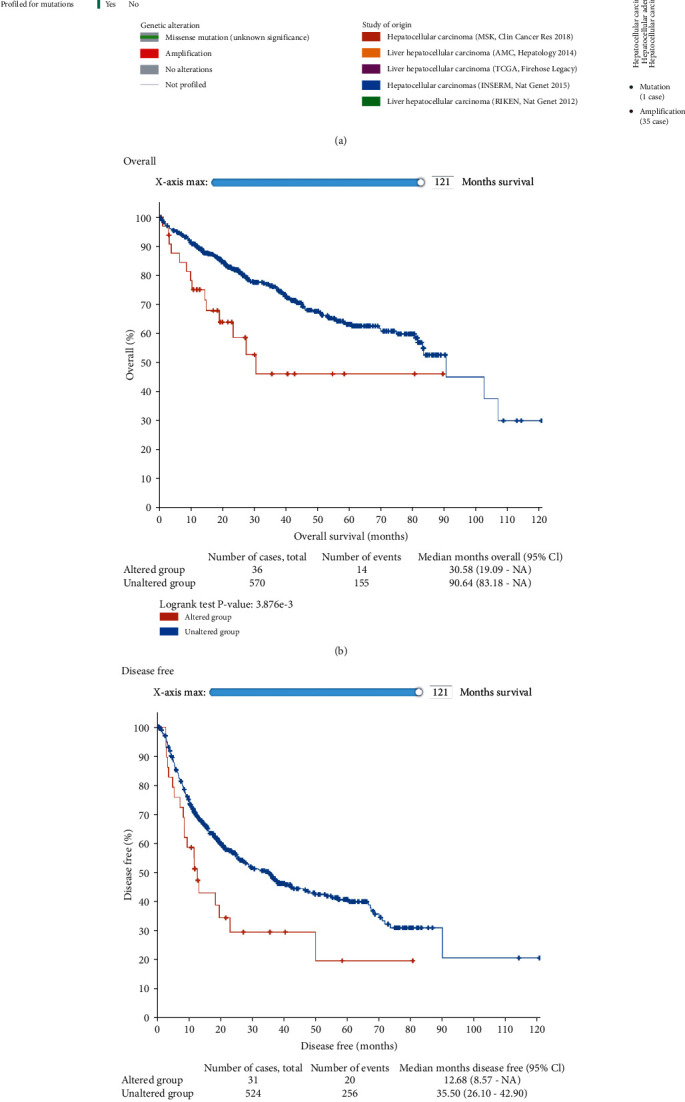
Mutation analysis of *CENPL* gene in HCC patients. Amplification was the most significant variation in 4% of the queried patients, and the study searched from 5 datasets (a). The gene variation was significantly associated with patient's OS and disease-free survival (*p* < 0.05) (b, c) (cBioPortal).

We used the cBioPortal database to investigate the mutations and copy number alterations of *CENPL*. Gene alterations occurred in 4% of the patients in the 5 datasets (Figure 7(a)). There was a significant correlation between the gene variation and patients' OS and disease-free survival (*p* < 0.05) (Figures 7(b) and 7(c)).

### 3.5. Relationship between the Expression of CENPL and Immune Cells in HCC

**Figure 8:**
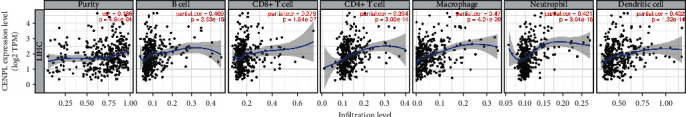
Relationship between the expression of *CENPL* and the abundance of immune cells in HCC. The expression of *CENPL* had positive correlations with tumor purity and significant positive correlations with infiltrating levels of B cells, CD4+ T cells, CD8+ T cells, neutrophils, macrophages, and dendritic cells in HCC, respectively (TIMER).

To further understand the relationship between *CENPL* mRNA level and immune infiltration cells in an HCC microenvironment, we used TIMER to evaluate immune cell infiltration data from The Cancer Genome Atlas (TCGA). The results illuminated that the expression of *CENPL* was positively correlated with infiltrating levels of B cell, CD4+ T cell, CD8+ T cell, neutrophil, macrophage, and dendritic cell in HCC, respectively (*p* < 0.01) (Figure 8). Similarly, the expression of the *CENPL* gene was positively associated with the tumor purity (*p* < 0.05) (Figure 8).

### 3.6. The Expression of *CENPL* Protein in Tumor and Normal Tissues of HCC Patients

**Figure 9:**
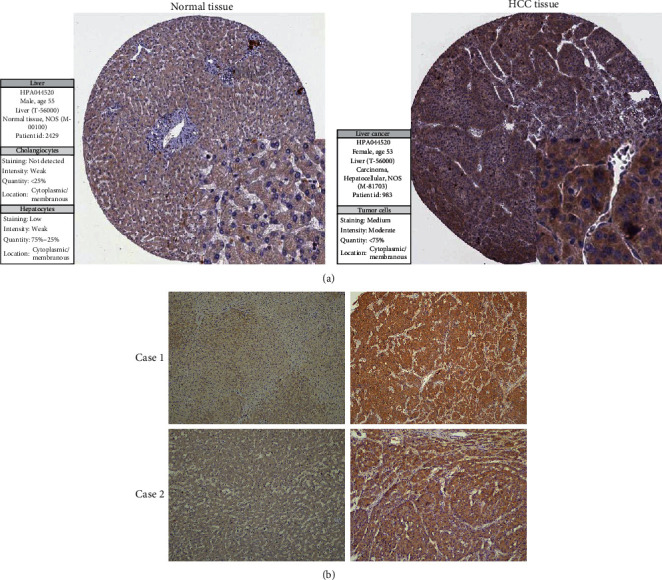
The expression of CENPL protein in tumor and normal tissues. Immunohistochemical staining showed that the expression of CENPL protein in HCC tissues was significantly higher than that in normal liver tissues. The CENPL protein was mainly localized in the cytoplasm and membrane (a) (HPA). In clinical specimens, the expression of CENPL protein in HCC tissues was significantly higher than that in adjacent tissues (b).

In order to learn the expression of CENPL protein in HCC specimens, we firstly studied it in the HPA database. The immunohistochemical staining results indicated that the expression of CENPL protein was significantly higher in HCC tissues than in normal liver tissues, and the protein staining intensity score was modest in HCC tissues and low in normal liver tissues (Figure 9(a)). The CENPL protein was mainly localized in the cytoplasm and membrane (Figure 9). Our further clinical specimens confirmed similar results. In 28 clinical specimens, the expression level of CENPL protein was higher in HCC tissues (16/28) than in noncancer tissues (12/28) (Figure 9(b)).

## 4. Discussion

In this study, the transcriptions of 15 CENPs in HCC were investigated by using the Oncomine and GEPIA databases. Results showed that the mRNA levels of *CENPL*, *CENPQ*, *CENPR*, and *CENPU* in HCC tissues were significantly higher compared with those in normal tissues. Their mRNA levels were significantly correlated with the pathological stages of HCC. Patients with higher mRNA levels of *CENPL* had poorer OS, PFS, RFS, and DSS. Then, the prognostic value of *CENPL* was further studied under different clinical parameters. We found that higher levels of *CENPL* mRNA were associated with poorer OS in white and Asian male patients without hepatitis virus infection. Mutation analysis showed that *CENPL* was rarely mutated, suggesting that the pathophysiological role of *CENPL* in HCC was not mediated by gene mutation. In addition, we found that the expression of *CENPL* was positively correlated with infiltrating levels of B cell, CD4+ T cell, CD8+ T cell, neutrophil, macrophage, and dendritic cell in HCC. Finally, HPA data and immunohistochemical staining of our clinical specimens showed that CENPL was overexpressed in HCC tissues.

CENPs play an important physiological role in the cell cycle, and their expression levels are strictly regulated; therefore, no pathological effect was shown. However, many CENPs with an abnormal expression were found in tumor cells. To date, it is not clear whether this result is an initiating factor or a result of tumor development. On the one hand, the abnormal expression of CENPs may interact with other genes and contribute to tumorigenisis. For example, Xiao et al. found that *CENPM* may be involved in the P53 pathway in HCC [40]. On the other hand, chromosomal and genetic instability are now recognized as the important cause of cancer, and the abnormal expression of CENPs is likely to play important roles through this mechanism [5, 41–43]. These results illustrate the complex mechanism between CENPs and HCC. Furthermore, the expression of CENPs in patients with cirrhosis has not been reported, which may help to reveal the mechanism of CENPs and HCC. *CENPL* is a subunit of the CENPH-CENPI-associated centromeric complex that targets *CENPA* to centromeres and is required for proper kinetochore function and mitotic progression [44]. Diseases associated with *CENPL* include the chromosome 22Q11.2 deletion syndrome and chromosome 17p13.3 duplication syndrome (GeneCards: https://www.genecards.org/cgi-bin/carddisp.pl?gene=CENPL&keywords=cenpl). To date, no studies of CENPL in tumors have been reported. In this study, we found that *CENPL* was overexpressed in HCC, and the mRNA level was closely related to various clinicopathological parameters of patients. These results suggest that CENPL may play an important role in HCC and that CENPL is a potential target for future immunotherapy and a prognostic predictor. However, there are several limitations to this study. We have not yet verified the association between *CENPL* and tumor-infiltrating lymphocytes in human or mouse specimens. The effects of *CENPL* on the proliferation, apoptosis, and metastasis of HCC cells have not been further studied. Therefore, in-depth studies are needed in the future.

In conclusion, the present study suggests that *CENPL* mRNA may be a potential prognostic biomarker of HCC patients.

## Figures and Tables

**Table 1:** Significant changes in the expression of CENPs in HCC and normal tissues (Oncomine database).

CENPs	Datasets	Fold change	*p* value	*t*-test
CENPL	Wurmbach et al.'s liver statistics	2.234	9.45*E*‐8	6.414
CENPQ	Chen et al.'s liver statistics (adenoma)	2.035	3.83*E*‐6	5.747
	Chen et al.'s liver statistics	2.458	1.18*E*‐10	6.934
CENPR	Roessler et al.'s liver 2 statistics	2.604	2.82*E*‐62	20.059
	Roessler et al.'s liver statistics	2.235	4.44*E*‐6	5.455
CENPU	Roessler et al.'s liver 2 statistics	4.019	2.46*E*‐66	22.356
	Chen et al.'s liver statistics	2.344	5.94*E*‐15	8.583
	Roessler et al.'s liver statistics	4.861	1.01*E*‐8	7.825
CENPV	Wurmbach et al.'s liver statistics	-2.392	7.60*E*‐7	-6.703
	Wurmbach et al.'s liver statistics	-2.075	4.18*E*‐6	-6.889

**Table 2:** Correlationship between the mRNA expression of CENPs and patient's survival (the Kaplan-Meier Plotter).

CENPs	Survival	Cases	HR	*p* value	Median survival	
					L (months)	H (months)
CENPL	OS	364	1.8	0.0009	70.5	38.3
	RFS	313	1.47	0.0218	34.4	21.2
	PFS	366	1.55	0.0036	29.77	13.13
	DSS	357	1.94	0.0033	49.67	20.9

CENPQ	OS	364	1.52	0.0186	70.5	45.7
	RFS	313	1.26	0.1722^∗^	30.4	21.23
	PFS	366	1.45	0.0136	29.77	13.83
	DSS	357	2.5	8.4*E*‐5	54.13	22

CENPR	OS	364	1.31	0.1205^∗^	70.5	52
	RFS	313	1.55	0.0095	37.67	17.9
	PFS	366	1.57	0.0024	36.1	15.07
	DSS	357	1.55	0.0538^∗^	84.4	104.17

CENPU	OS	364	1.31	0.1243^∗^	70.5	49.7
	RFS	313	1.67	0.0021	37.67	16.37
	PFS	366	1.62	0.0012	33	15.07
	DSS	357	1.37	0.161^∗^	81.87	84.73

*Note*. L: low-expression cohort; H: high-expression cohort. “∗” means no significant statistical significance, *p* > 0.05.

**Table 3:** The relationship between the mRNA expression of CENPL and patients' OS with various clinical parameters (the Kaplan-Meier Plotter).

Gender	Race	Risk factors (yes/no)		Cases	HR	*p* value	Median survival	
Male*/*female	White/Asian	Alcohol consumption	Hepatitis virus				L (months)	H (months)
Both	All	Both	Both	364	1.8	0.0009	70.5	38.3
Male	All	Both	Both	246	2.17	0.0007	82.9	36.3
Female	All	Both	Both	118	1.02	0.9349^∗^	52	46.6
Both	White	Both	Both	181	1.55	0.0599^∗^	54.1	31
Both	Asian	Both	Both	155	3.36	0.0002	56.2	9.9
Both	All	Yes	Both	115	3.31	0.0003	81.9	26.7
Both	All	No	Both	202	1.64	0.0367	71	38.3
Both	All	Both	Both	150	1.17	0.632^∗^	54.1	23.1
Both	All	Both	No	167	3.61	5.0*E*‐7	70.5	22
Male	White	Both	Both	102	2.28	0.0174	54.1	27.9
Male	Asian	Both	Both	122	3.72	0.0003	56.2	9.3
Male	White	Both	Yes	32	1.33	0.6475^∗^	41	36.3
Male	White	Both	No	54	2.99	0.0224	59.7	27.9
Male	Asian	Both	Yes	77	1.37	0.5562^∗^	NA	NA
Male	Asian	Both	No	37	3.97	0.0098	17.8	5.7

*Note*. L: low-expression cohort; H: high-expression cohort. “∗” means no significant statistical significance, *p* > 0.05.

## Data Availability

The datasets analyzed during the current study are available from Oncomine (http://www.oncomine.org), GEPIA (http://gepia.cancer-pku.cn), the Kaplan-Meier Plotter (http://www.kmplot.com), cBioPortal (https://www.cbioportal.org), TIMER (https://cistrome.shinyapps.io/timer/), and HPA (https://www.proteinatlas.org/).
